# Dissecting a Role for Melanopsin in Behavioural Light Aversion Reveals a Response Independent of Conventional Photoreception

**DOI:** 10.1371/journal.pone.0015009

**Published:** 2010-11-29

**Authors:** Ma'ayan Semo, Carlos Gias, Ahmad Ahmado, Eriko Sugano, Annette E. Allen, Jean M. Lawrence, Hiroshi Tomita, Peter J. Coffey, Anthony A. Vugler

**Affiliations:** 1 Department of Ocular Biology and Therapeutics, University College London-Institute of Ophthalmology, London, United Kingdom; 2 Institute for International Advanced Interdisciplinary Research, Tohoku University, Aoba-ku, Sendai, Japan; 3 Faculty of Life Sciences, University of Manchester, Manchester, United Kingdom; Dalhousie University, Canada

## Abstract

Melanopsin photoreception plays a vital role in irradiance detection for non-image forming responses to light. However, little is known about the involvement of melanopsin in emotional processing of luminance. When confronted with a gradient in light, organisms exhibit spatial movements relative to this stimulus. In rodents, behavioural light aversion (BLA) is a well-documented but poorly understood phenomenon during which animals attribute salience to light and remove themselves from it. Here, using genetically modified mice and an open field behavioural paradigm, we investigate the role of melanopsin in BLA. While wildtype (WT), melanopsin knockout (*Opn4^−/−^*) and *rd/rd cl* (melanopsin only (MO)) mice all exhibit BLA, our novel methodology reveals that isolated melanopsin photoreception produces a slow, potentiating response to light. In order to control for the involvement of pupillary constriction in BLA we eliminated this variable with topical atropine application. This manipulation enhanced BLA in WT and MO mice, but most remarkably, revealed light aversion in triple knockout (TKO) mice, lacking three elements deemed essential for conventional photoreception (*Opn4^−/−^ Gnat1^−/−^ Cnga3^−/−^*). Using a number of complementary strategies, we determined this response to be generated at the level of the retina. Our findings have significant implications for the understanding of how melanopsin signalling may modulate aversive responses to light in mice and humans. In addition, we also reveal a clear potential for light perception in TKO mice.

## Introduction

In the 1920's, Crozier and Pincus showed that neonatal rats with closed eyelids will move away from bright light along a gradient towards a less intensely illuminated target [1]. Adult rats retain an aversion to light [2], [3], so strong that it can be used as a motivating factor in behavioural learning paradigms [4]. Like rats, adult mice also show behavioural light aversion (BLA) to acute (10–30 mins) light exposure in the open field [5], [6]. Using mice, the “light/dark box test” has been employed extensively in drug development to identify putative anxiolytic compounds (see [5], reviewed in [7]) and more recently to investigate human photophobia in mouse models of migraine [8], [9]. Despite the widespread application of this behavioural phenomenon, and its undoubted importance to the lives of nocturnal animals, little is known about the neural circuitry mediating BLA in rodents. Although one study to date has implicated both subcortical and cortical processing [10], the contribution of different photoreceptive components from the retina remains unclear.

In the mammalian retina, rods/cones of the outer retina are known to mediate image-forming vision [11], [12], while photoreceptive melanopsin-expressing retinal ganglion cells (mRGCs) of the inner retina sub serve most non-image-forming responses to light [13], [14], [15], [16], [17]. If the eyes are enucleated bilaterally, then BLA in rats is abolished [10]. To date, only a few studies shed light on the important question of whether melanopsin alone could mediate this primitive non-image-forming response. These studies, from a variety of animal models, report mixed conclusions about a potential role for melanopsin in BLA.

A recent study investigating the role of melanopsin in non-image forming functions found that targeted destruction of melanopsin cells had no impact on the light:dark preference of mice [18]. This is in line with data from RCS rats showing a progressive loss of BLA over time, with no response detectable by 7 months [19]. Another study using *rd* mice also failed to report a significant light aversion response following exposure to illumination of 2800 Lux [20].

In contrast, spatial responses to light have been reported in *rd* mice given the choice between light and dark living/nesting areas over a 22 h period [21]. Here, retinal degenerate mice spent significantly more time in the dark than the illuminated area, a response that could be eliminated by enucleation. However, as *rd* mice retain a significant population of remodelled cones with identifiable presynaptic stuctures [22], [23], [24], [25] they are unsatisfactory for defining a role for melanopsin in BLA. In the present study we employ the *rd/rd cl* mouse, which lacks both rods and cones [15].

Melanopsin is a retinaldehyde-based, invertebrate-like photopigment [26], [27] involved with mediating many responses to light that require a measure of general environmental irradiance [14], [15], [28], [29], [30] and more recently, the ability of light to modulate sleep [31], [32], [33]. Importantly, an associative learning (Pavlovian conditioning) paradigm has shown that *rd/rd cl* mice can gradually learn to use a brief light stimulus to predict the onset of electric shocks [34]. Although melanopsin cells are thought to project mainly to subcortical, non-image forming centres of the brain, they may also signal luminance information to the visual cortex [35], [36], [37], [38].

In humans, light aversion is often referred to as photophobia, a clinical term describing pain onset following light exposure in a number of conditions including migraine headache [39], [40], [41]. Recently, the melanopsin system has been implicated in the potentiation of migraine by light in blind patients [42] and although little is known about the neural circuitry of photophobia it is generally considered to require a convergence of information from optic and trigeminal nerves with associated cortical processing [40], [42], [43], [44]. In addition, because sensory trigeminal afferents innervate muscles of the iris, sustained constriction caused by the pupillary light reflex (PLR) has also been implicated in causing the ocular discomfort felt following exposure to bright lights [40], [41], [45]. The term photophobia is also used to describe the sensation felt when we, as humans, enter an environment which is subjectively appraised as being “too bright”, eliciting aversive behavioural responses such as looking away from bright light and squinting [46], [47], [48].

Our goal in the present study was to determine the extent to which melanopsin mediates BLA in mice. To achieve this, we developed a variation on an established protocol for measuring light aversion in mice [6], which now takes into account the behaviour of animals placed into complete darkness. We tested naïve wildtype (WT) mice, *rd/rd cl* mice (hereafter referred to as melanopsin only (MO)) [14], [31], melanopsin knockout (MKO) mice (*Opn4^−/−^*) [30] and as a control for the absence of light perception, triple knockout (TKO) mice, lacking melanopsin and functional rods/cones (*Opn4^−/−^ Gnat1^−/−^ Cnga3^−/−^*). These mice have no significant PLR, circadian photoentrainment or masking responses [49]. In order to investigate if pupillary constriction is causally related to BLA, we also equalised this variable across genotypes by applying atropine bilaterally to the eyes.

Our experiments show that melanopsin alone can mediate a behavioural aversion to light that is associated with neural activation in the extended visual cortex. Analysis of temporal kinetics reveals that melanopsin acts slowly to increase light aversion over time, whereas rods/cones drive a more immediate aversive response. While MKO mice remain capable of BLA our analysis reveals that rods/cones and melanopsin are required for an aversive response characteristic of WT animals. Surprisingly, the addition of atropine increased BLA in WT, MO and TKO mice, with this new light perception in TKO's being associated with an enhancement of residual retinal activity. The retinal origin of light aversion behaviour in TKO mice was further investigated by either eliminating BLA with bilateral axotomy or generating a response comparable to that seen in wildtype animals by specifically activating retinal neurons using *Channelrhodopsin-2*.

## Results

### Melanopsin alone can drive the behavioural aversion to light

**Figure 1:**
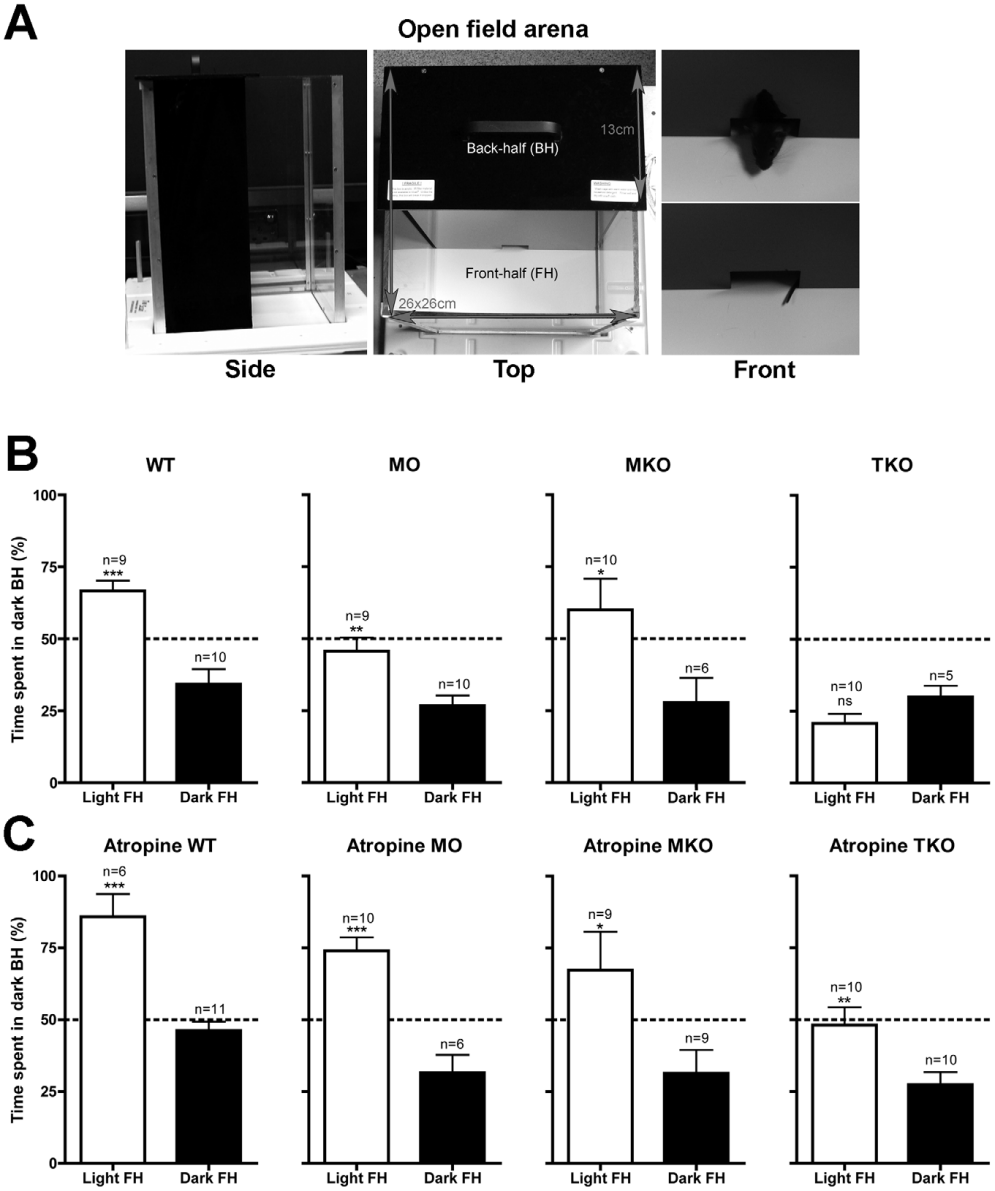
Role of melanopsin in the behavioural aversion to light in mice. (**A**) Open field apparatus: animals were placed into the front-half (FH) of the arena and remained there for 30 minutes. Time spent in the back-half (BH) of the arena was recorded. (B) and (C) Average (±SEM) percentage of time spent in the dark BH of the arena during the 30-minute trial. The FH is either illuminated, white bars (light FH), or in darkness, black bars (dark FH). (**B**) In untreated animals photophobic behaviour is evident in wildtype (WT), melanopsin only (MO) *rd/rd cl* mice, and melanopsin knockout (MKO) mice. Triple knockout (TKO) mice, lacking melanopsin and functional rods and cones show no aversion to light. (**C**) Atropine significantly increases aversive behaviour in WT, MO, and TKO mice. In MKO mice, atropine increases the average aversive behaviour but this does not reach significance. Atropine does not significantly affect behaviour when the FH is in darkness in any of the genotypes. Stars (*) indicate significance levels (Student's *t*-test): * *p*<0.05; ** *p*<0.01; *** *p*<0.001.

Animals were tested for BLA for 30 minutes in the open field apparatus shown in Figure 1A. This behaviour was assessed by comparing time spent in the dark back-half (BH) when the front-half (FH) was illuminated (light FH) with control conditions when the FH was maintained in darkness (dark FH).

Over the whole trial, WT normally-sighted animals spend the majority of their time (67%) in the dark BH of the arena when the FH is illuminated (Figure 1B). This is also significantly more time (*p*<0.001) than when the FH is maintained in darkness during which they spend only 34% of the time in the BH. The *rd/rd cl* animals, with only melanopsin as a functional photopigment (MO) do not spend the majority of their time in the dark when the FH is illuminated (46%), however a significant light-aversion response is revealed when this is compared to the amount of time that is spent in the BH when there is no illumination (27%) (*p*<0.01).

This result, together with previous observations of an impairment to BLA following lesions of visual cortex [10] prompted us to examine if melanopsin alone could drive activation of this structure in mice. This was achieved by examining light-induced c-Fos in the visual cortex of MO animals, a technique previously validated for normally sighted mice [50]. Here, using the same light source as that used for behavioural testing, we found a clear, melanopsin-driven c-Fos induction in medial visual/retrosplenial cortex (Figure S1).

### Melanopsin is not required for the behavioural aversion to light

As seen in Figure 1, rods/cones also play a major role in BLA. When the behaviour of the congenic MO and WT mice are compared by two-way ANOVA there is a significant effect of genotype (*p*<0.01) and of light (*p*<0.0001), with Bonferroni's multiple comparison tests confirming a significant reduction in time spent in the dark BH when the FH is illuminated in the MO (46%) compared to the WT (67%) mice (*p*<0.01). In control conditions, when the entire arena is in darkness there is no significant difference in behaviour between MO and WT mice, both seeming to retain a preference for the FH.

Animals lacking melanopsin (MKO) spend significantly more time (60%, *p*<0.05) in the dark BH when light is on in the FH (Figure 1B), than when there is no illumination (only 28% of time spent in BH). This finding shows that although melanopsin alone can mediate BLA, the presence of this photopigment is not a requirement for this response to occur. As anticipated, in TKO mice (lacking melanopsin and normal rod/cone function), there is no response to illumination in the FH, with these mice spending similar amounts of time (*p*>0.05) in the BH whether the FH is in darkness or light (29 versus 21% of the time).

Interestingly, regardless of whether the light was on or off, TKO mice spend most of their time in the open FH of the arena, as do the other genotypes in the complete darkness control condition. This phenomenon holds true regardless of which side of the arena animals are first placed (data not shown). To the best of our knowledge, this consistent behaviour has not been reported previously and should be taken into account when interpreting data derived from light:dark choice tests of a similar design to ours.

### Temporal kinetics of light aversion in mice

**Figure 2:**
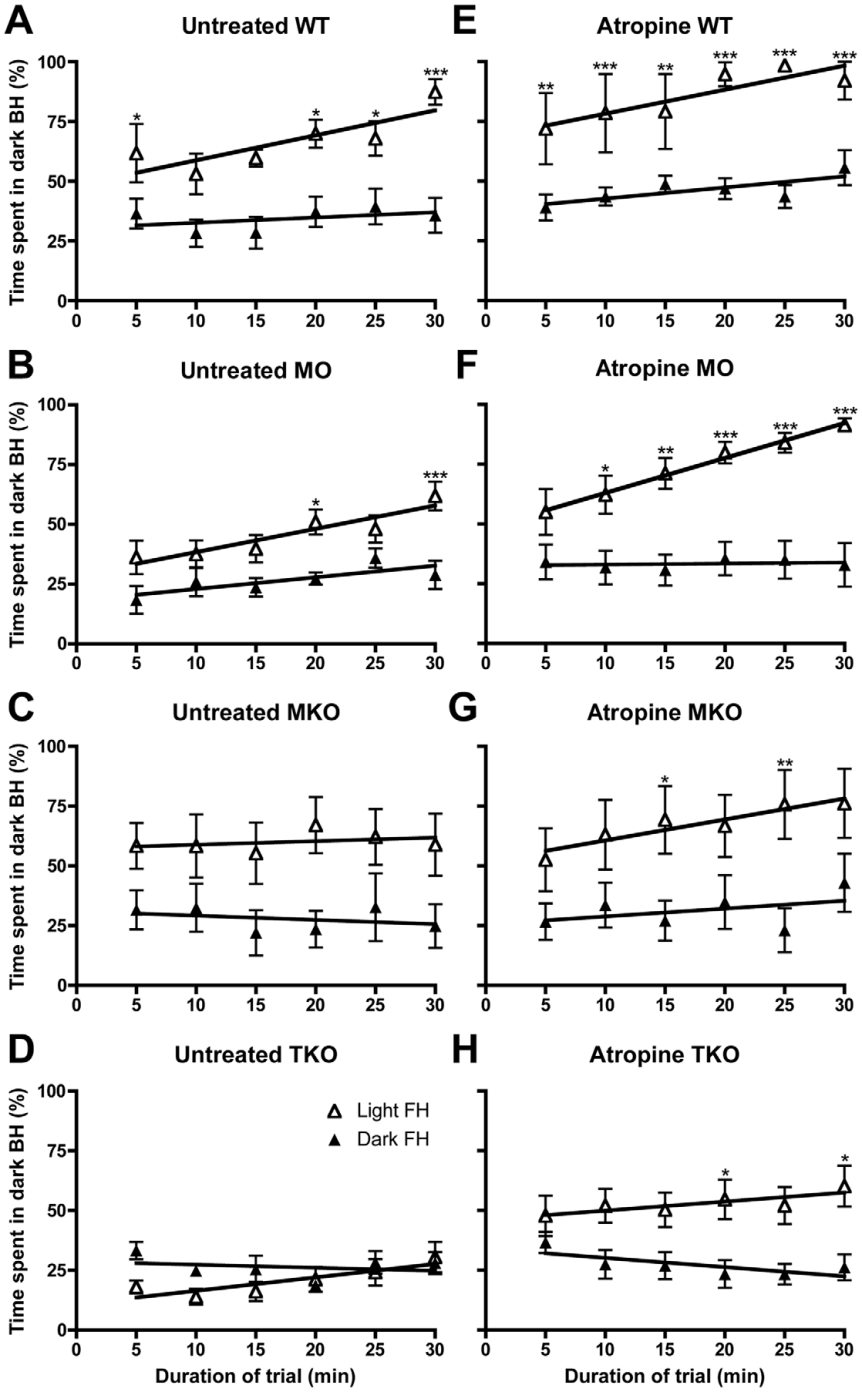
Temporal kinetics of the behavioural aversion to light in mice. Graphs showing time spent in the dark back-half (BH) of the arena over the course of the 30-minute trial. Data are binned into 6, 5-minute bins throughout the trial, with y-axis showing average (±SEM) percentage time spent in the dark BH. (**A–D**) shows data from untreated animals, and (**E–H**) after bilateral application of atropine drops. (**A**) and (**E**) WT, (**B**) and (**F**) MO (*rd*/*rd cl*), (**C**) and (**G**) MKO (*Opn4^−/−^*) and (**D**) and (**H**) TKO (*Opn4^−/−^ Gnat1^−/−^ Cnga3^−/−^*). White triangles, trials when the front-half (FH) is in light, black triangles, trials when the FH is in darkness. Results of the regression analyses are shown in table 1. Stars (*) indicate significance levels (Bonferroni post tests, light FH v dark FH at each time point): * *p*<0.05; ** *p*<0.01; *** *p*<0.001.

To investigate the behaviour of mice during the course of the 30-minute trial, data were binned into 6, 5-minute bins throughout the trial (Figure 2A–D). Results of the associated regression analysis are summarised in table 1. Under control conditions (complete darkness), in all genotypes, animals failed to change the amount of time spent in the BH (slopes of regression lines are not significantly non-zero). However, when there is light in the FH of the arena, both WT and MO mice show a positive correlation with duration of the trial, spending more time in the dark BH in the last 5 minutes. To compare the effect of the light to control conditions over the course of the trial, two–way repeated measures ANOVA (RM ANOVA) was carried out on data from each genotype, the results of Bonferroni post-tests are indicated on the graphs in Figure 2.

**Table 1:** Regression analysis of temporal kinetics of light aversion in mice.

Genotype	Treatment	Significant non-zero slope (*p*)	r^2^	Slope
WT	Light Untreated	<0.05	0.68	1.05±0.36
WT	Dark Untreated	>0.05	-	-
MO	Light Untreated	<0.01	0.86	0.97±0.20
MO	Dark Untreated	>0.05	-	-
MKO	Light Untreated	>0.05	-	-
MKO	Dark Untreated	>0.05	-	-
TKO	Light Untreated	<0.05	0.75	0.56±0.16
TKO	Dark Untreated	>0.05	-	-
WT	Light Atropine	<0.05	0.78	1.01±0.27
WT	Dark Atropine	>0.05	-	-
MO	Light Atropine	<0.0001	0.99	1.46±0.07
MO	Dark Atropine	>0.05	-	-
MKO	Light Atropine	<0.01	0.87	0.88±0.17
MKO	Dark Atropine	>0.05	-	-
TKO	Light Atropine	<0.05	0.70	0.38±0.12
TKO	Dark Atropine	>0.05	-	-
TKO	Light Axotomy/Atropine	>0.05	-	-
TKO	Dark Axotomy/Atropine	>0.05	-	-
TKO	Light AAV2-ChR2V	<0.05	0.66	0.78±0.28

The WT (Figure 2A) and MO (Figure 2B) mice show a similar pattern of behaviour over the course of the trial, with the RM ANOVA test revealing a significant effect of time (WT *p*<0.01, MO *p*<0.001), light (WT *p*<0.001, *p*<0.01) and an interaction between time X light (WT *p*<0.05, MO *p*<0.05). In the last 5 minutes of the trial (minutes 25–30) the WTs and MOs spend the highest proportion of their time in the dark (87% WTs and 62% MOs). It is however clear when comparing the behaviour of WTs and MOs that melanopsin does not mediate all aspects of normal light aversion behaviour. Unlike MOs, the WT mice show a significant aversive response during the first 5 minutes of the trial and spend a higher proportion of their time in the dark BH.

As shown in Figure S2, aged MO animals retain their BLA, despite a well-documented loss of melanopsin cells with advancing age in these animals [51], [52]. Interestingly, aging alters the behaviour of MO mice over the duration of the trial, so that during the first 5 minutes of the trial light aversion is intensified in older MOs compared to younger animals (Figure S2B). This result is of note and implies an increase in the potency of melanopsin signalling in retinal dystrophy with advancing age.

In MKO mice we found no significant correlation with duration of the trial and time spent in the dark BH. From the beginning to the end of the trial these mice spent 60% of their time in the back-half, similar to their overall average (Figure 2C). For this group of mice RM ANOVA showed a significant effect of light (*p*<0.05), but not for duration of trial or the interaction term light X time. As expected, the TKO mice did not display a significant aversion to light, with RM ANOVA showing no significant effect of light, time or the interaction term, with no differences between light FH and dark FH by Bonferroni post-tests. However, rather curiously, over the 30-minute trial duration, TKO mice do show a positive correlation with respect to the amount of time spent in the dark BH of the arena when the FH is illuminated (see Figure 2D and table 1).

In summary, when rods and cones are absent, melanopsin is capable of driving a slower onset BLA that is only clearly revealed after 15–20 minutes. Conversely, in the absence of melanopsin, animals retaining significant light aversion lack the positive correlation over time. Animals lacking melanopsin and properly functioning rods and cones (TKO) do not exhibit significant light aversion. Therefore, in order to display the aversion to light characteristic of their species, rodents must possess rods/cones and the photopigment melanopsin.

### Ocular application of atropine enhances light aversion

In order to investigate the impact of eliminating the variable of pupillary constriction on BLA, atropine drops were applied bilaterally to the eyes 30 minutes prior to placing naïve animals into our open field arena. Other mydriatics were tested initially (e.g. phenylephrine, and tropicamide), however these agents were found to be either too short acting for the 30-minute trial and/or to cause mild distress, as such they were deemed unsuitable for use in combination with behavioural testing. Atropine on the other hand was ideal for this experiment as it relaxes the circular muscles of the iris to cause a painless and long-lasting mydriasis [53].

**Figure 3:**
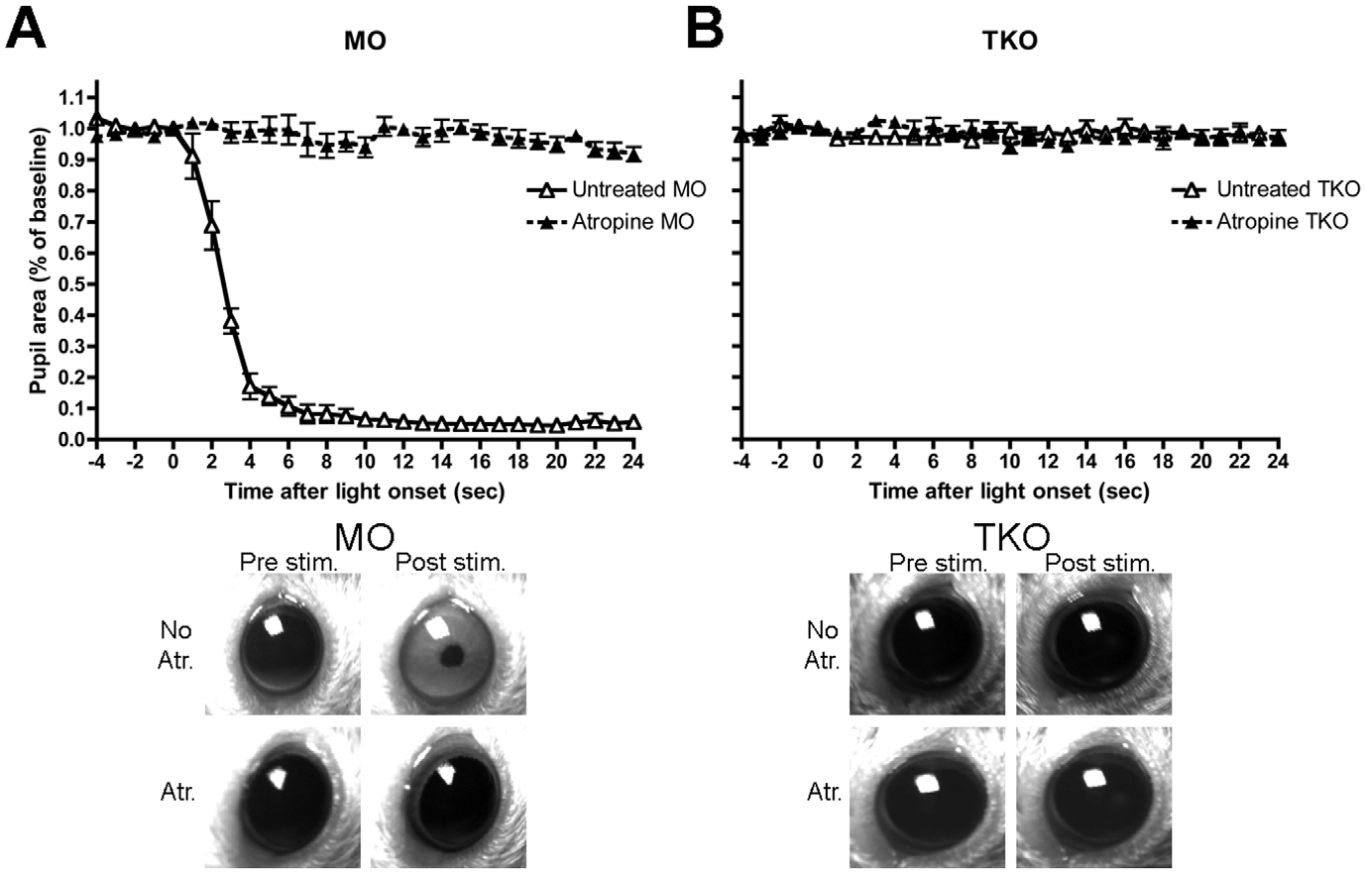
Effect of atropine on pupil size. In (**A**) MO (*rd/rd cl*), and (**B**) TKO (*Opn4^−/−^ Gnat1^−/−^ Cnga3^−/−^*) mice. Images below each graph illustrate pupil size pre- and post- light stimulation with atropine (Atr.) or without atropine (No Atr.) application in the two genotypes.

The application of atropine to the eyes of experimental animals produced no outward signs of discomfort and resulted in sustained pupil dilation. Figure 3A shows the PLR of MO mice to white light illumination (intensity-matched to that found in the experimental arena), following atropine application, the pupil no longer constricts. Unlike the other three genotypes tested here, TKO mice already lack pupil constriction, with neither atropine nor illumination able to change their pupil area (Figure 3B). It should be noted that the constriction mechanism itself in TKO mice remains intact, as demonstrated previously by application of the parasympathetic agonist carbachol [49].

As shown in Figure 1C, when atropine is applied prior to testing, all genotypes (including TKO mice) exhibit significant BLA, spending more time in the dark BH when the FH is illuminated. The influence of light and atropine in each genotype was assessed by two-way ANOVA followed by Bonferroni's multiple comparison tests. In the WT and MO there is a significant effect of light (WT, *p*<0.001; MO *p*<0.001) and atropine (WT, *p*<0.001; MO, *p*<0.01), there is also a significant interaction between light X atropine only in MO (WT, not significant; MO *p*<0.05). *Post hoc* tests reveal that with atropine application, light aversion is significantly increased (*p*<0.01) from 67% to 86% in WTs, and from 46% to 74% (*p*<0.001) in MOs. Atropine did not have a significant effect on behaviour in the dark in either the WTs or MOs.

Comparing the WT and MO behaviour by two-way ANOVA (factors: light and genotype) there is still an effect of both light (*p*<0.0001) and genotype (*p*<0.05) but by *post hoc* comparison testing the MO is no longer significantly less light aversive than the WT as was the case in the non atropine treated animals. Atropine application is therefore greatly enhancing melanopsin- mediated BLA, such that MOs lacking rods and cones are now behaving much more like WT animals. By contrast, in MKO mice atropine does not significantly enhance light aversion (Figure 2C), with two-way ANOVA revealing a significant effect of light (*p*<0.01) but not of atropine. In these animals, *post hoc* testing confirms there is no statistically significant change in light aversion with atropine application when either the FH is illuminated or in control conditions when the entire arena is in darkness.

Rather surprisingly, two-way ANOVA reveals a significant effect of atropine (*p*<0.05) and a significant interaction between light and atropine (*p*<0.05) in the TKO mice. *Post hoc* testing confirms that there is a significant increase in light aversion behaviour with the application of atropine (*p*<0.01) from 21% to 53% of time spent in the dark BH when the light is on. Again, atropine had no influence on behaviour in control conditions when the FH is in darkness.

Following atropine application, all the genotypes now show a positive correlation over the course of the trial spending more time in the dark BH as the trial progresses when the FH is illuminated (Figure 2E–H; table 1). In the last 5 minutes of the light FH trial, WT mice spend 92% of the time in the dark BH, and at this point the MO is almost indistinguishable, spending 91% of the time in the dark. The MKO also spends most time in the dark at this point (76%), and surprisingly the TKO also exhibits quite a striking aversion to light, spending 60% of the time in the dark in the final 5 minutes of the trial. Two–way RM ANOVA of atropine treated WT (Figure 2E), MO (Figure 2F), and MKO (Figure 2G), behaviour reveals a significant effect of time (WT, *p*<0.01; MO, *p*<0.0001; MKO, *p*<0.001) light (WT, *p*<0.001; MO *p*<0.001; MKO, *p*<0.001) and an interaction between time X light (WT, *p*<0.05; MO, *p*<0.01; MKO *p*<0.01). The two-way RM ANOVA on atropine treated TKO mice (Figure 2H) shows there to be a significant effect of light (*p*<0.05) and a significant interaction between light X time (*p*<0.05) on BLA. It is clear that towards the end of the trial, TKO mice now spend significantly more time in the dark BH when the light is on in the FH than in control conditions with the dark FH.

**Figure 4:**
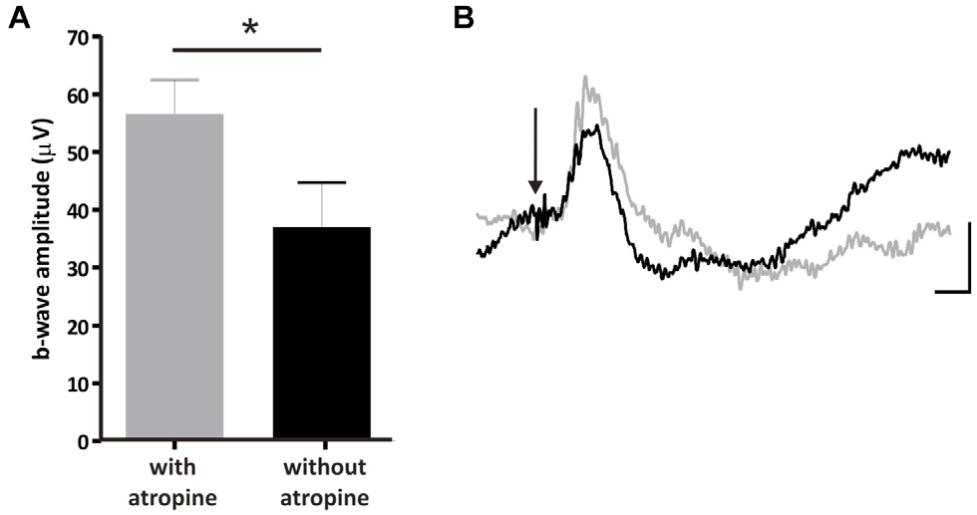
Atropine augments an ERG b-wave preserved in TKO mice. (**A**) b-wave amplitude of flash ERG responses in the presence and absence of atropine drops. A small but significant increase in the ERG b-wave amplitude was apparent following application of atropine drops (data presented as mean±SEM; n = 5 for each group). This is demonstrated in the average of all ERG responses in each group, shown in (**B**), atropine treated shown in grey, and untreated in black (scale bars: y-axis = 25 µV, x-axis = 50 ms; n = 5 for each group).

The mechanism by which atropine is increasing light aversion in TKO mice is not readily apparent. In the other three genotypes (WT, MO and MKO), atropine application is causing pupil dilation and as such, their enhanced behavioural response could be attributed to more light entering the eye. However, in TKO mice this cannot be the case as we found their pupils to be fully dilated regardless of atropine administration (Figure 3B). As such, atropine would appear to be enhancing some residual light perception retained in these animals. This may be at the level of the retina or, alternatively, through a more systemic route acting on more central components of the visual system. Indeed, recent work from the Lucas laboratory has identified a small but significant electrophysiological response to light, both at the level of the ERG and the dorsal lateral geniculate nucleus of the thalamus [54]. To test the hypothesis that atropine might be influencing responsiveness of the TKO retina directly we carried out ERG recordings on these mice with and without atropine. Atropine does indeed significantly enhance the b-wave amplitude in TKO mice (Figure 4).

### Aversion to light in TKO mice is driven by signals from the retina

In order to determine if the atropine-enhanced BLA of TKO mice was being driven by signals from the retina (as opposed to a local-systemic action of this drug on the brain), we used two complementary approaches: 1. Eliminate retinal input to the brain using bilateral axotomy and 2. Specifically render retinal neurons light sensitive using a non-pharmacological agent (the microbial opsin *Channelrhodopsin-2* (*ChR2*)), unable to potentiate the function of more remote components of the visual system.

**Figure 5:**
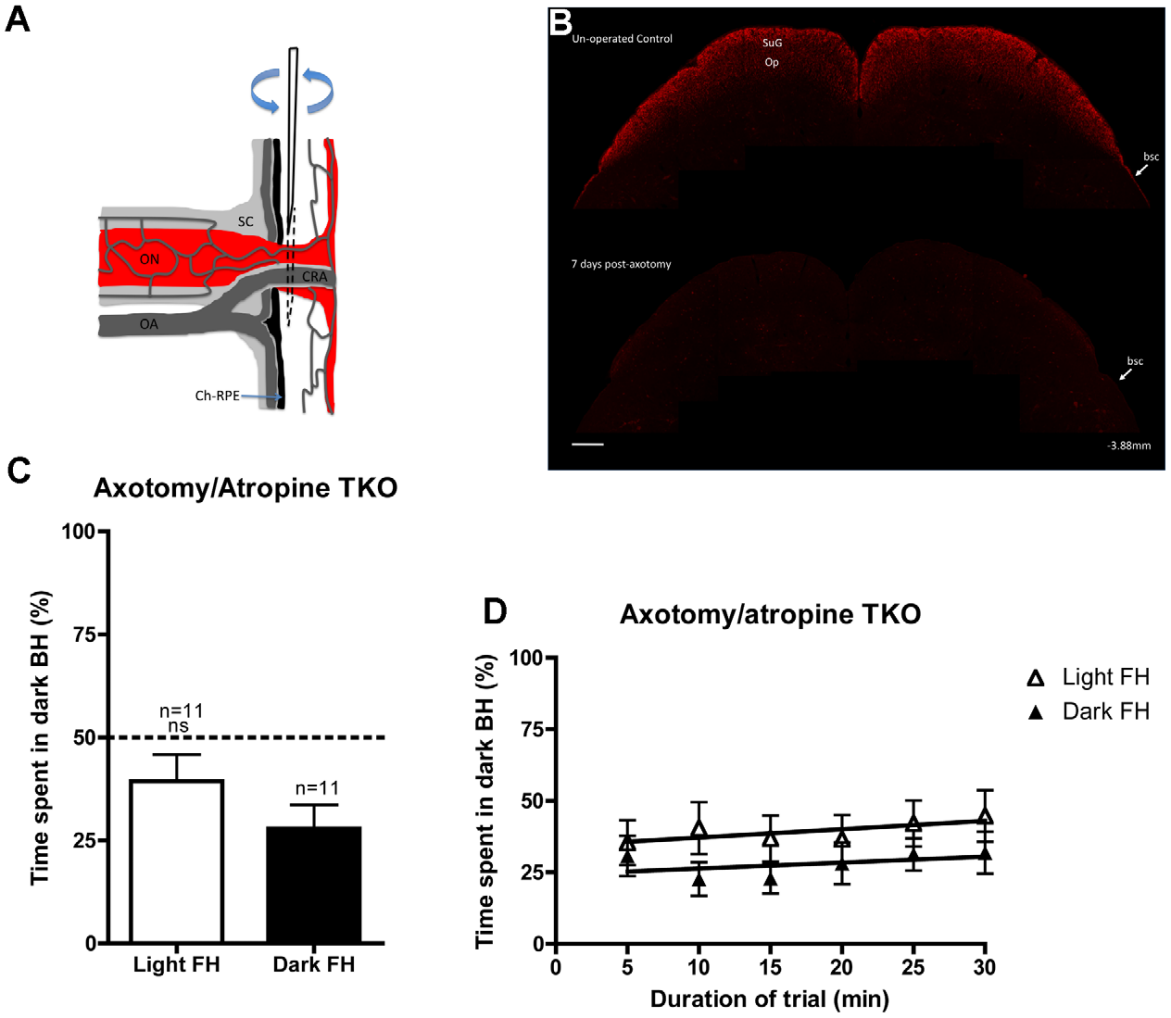
Axotomy abolishes the atropine-induced light aversion response in TKO mice. (**A**) Diagramatic illustration of the axotomy technique (image modified from [75] ), a swift back and forth movement of the needle severs both the optic nerve and central retinal artery. (**B**) Immunoreactivity for calretinin positive retinal afferents (red) is abolished in the superficial gray (SuG) and the optic nerve (Op) layers of the superior colliculus of a bilaterally axotomised TKO (bottom) compared to an unoperated contol (top). Collicular sections are −3.88 mm from bregma [76], scale bar is 200 µm. (**C**) Behavioural aversion to light in atropine-treated TKO mice is abolished in bilaterally axotomised animals. (**D**) Time spent in the dark back half (BH) of the arena over the course of the 30-minute trial. White triangles, from trials when the front-half (FH) is in light, black triangles when the FH is in darkness. Abbreviations: bsc, brachium of the superior colliculus; Ch-RPE, choroid retinal pigment epithelium; CRA, central retinal artery; ns, not significant; OA, ophthalmic artery, ON, optic nerve; Sc, sclera.

For axotomy, in order to minimise the trauma associated with established procedures [55], [56], we developed a novel technique that uses an intraocular, sub-retinal approach (see diagram in Figure 5A). As shown in Figure 5B and Figure S3, at 9 days post-axotomy, our technique has obliterated calretinin-positive retinal axons innervating the brain, confirming successful axotomy.

As shown in Figure 5C, after axotomy and subsequent atropine application, TKO mice no longer show a significant light aversion response over the whole trial (light FH versus dark FH Student's *t*-test *p*>0.05). Over the course of the trial there is also no correlation with the amount of time the animals spend in the dark BH with light FH or dark FH (see Figure 5D; Table 1). Also, Two-way RM ANOVA did not reveal any significant effects of light or trial duration on the time spent in the dark BH. These data show that axotomy abolishes the atropine-induced BLA in TKO mice.

In order to confirm that enhanced retinal output is sufficient to drive BLA in TKO mice, we rendered their retinae directly light sensitive. This was achieved by transfecting inner retinal neurons with *ChR2* using an intravitreal injection of an adeno-associated viral vector (AAV), which causes *Channelrhodopsin-2/Venus* (ChR2V) fusion protein expression in the retinal ganglion cells [57], [58]. The expression of *ChR2V* gene is under the control of the CAG promoter which results in approximately 30% of retinal ganglion cells expressing *ChR2* [58]. It has previously been demonstrated that the viral construct we use here (AAV2-*ChR2V*) restores visual responses in rodents with degenerate rods/cones, while the Venus fluorescent reporter alone (AAV2-*Venus*) does not [57], [58].

**Figure 6:**
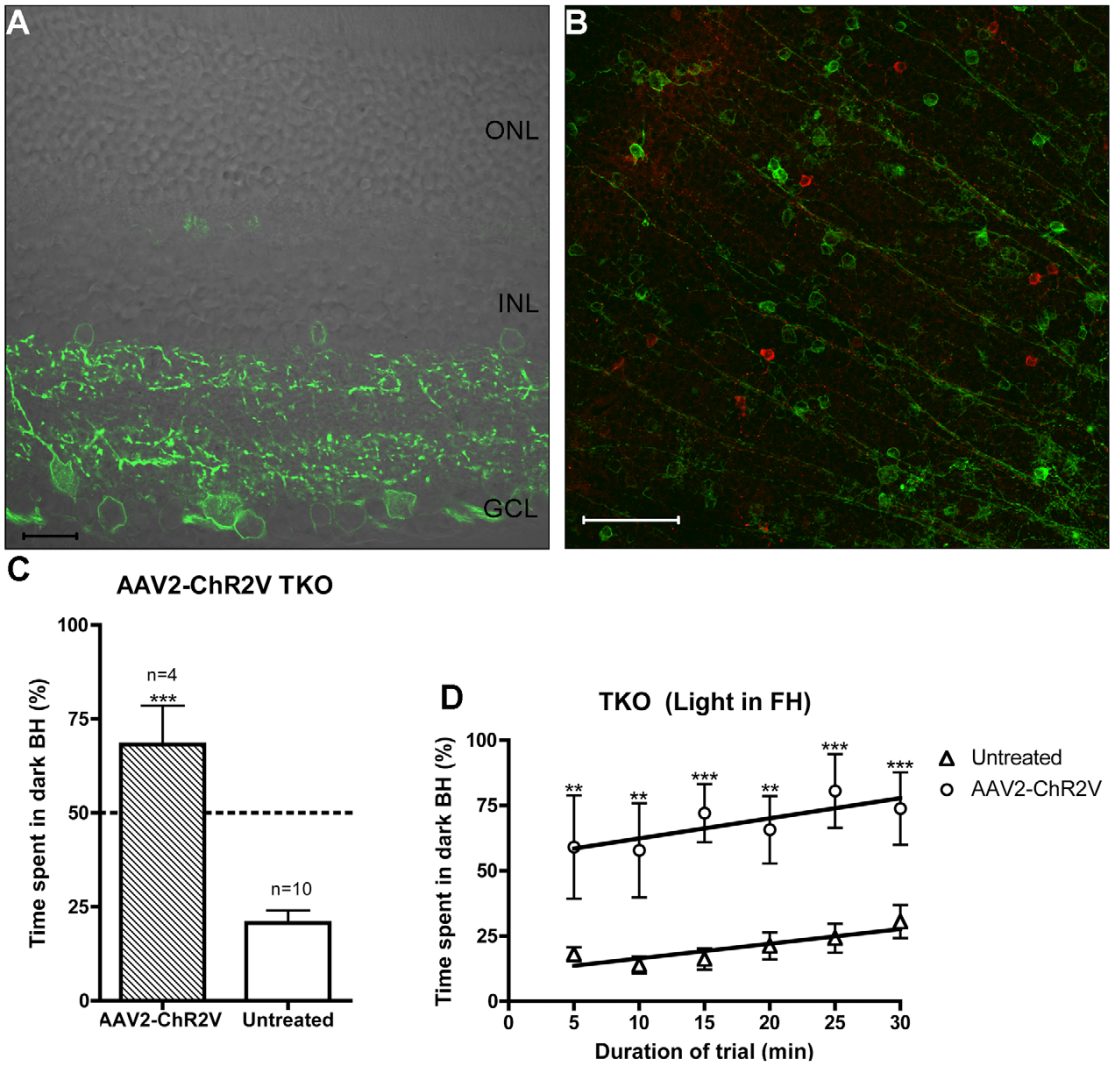
Channelrhodopsin-2 expression in the inner retina of TKO mice causes the induction of behavioural light aversion. (**A**) and (**B**) AAV2 transduced expression of Channelrhodopsin-2/Venus fusion (ChR2V) protein (green) in the ganglion cell layer of a TKO retina (AAV2-*ChR2V* TKO). (**A**) Transverse retinal section, ChR2V is visualised in many cells of the ganglion cell layer. Scale bar 20 µm (**B**) Immunohistochemistry on flat mount retina (focussing on the ganglion cell layer) for β-galactosidase (red) with ChR2V in green. Scale bar 100 µm. (**C–D**) Light aversion behaviour in the AAV2-*ChR2V* TKO. In these two graphs the comparison is between transduced and untreated animals when there is illumination in the front-half (FH). (**C**) Time spent in the dark back-half (BH) of the arena during the total 30 minutes of the trial. (**D**) Time spent in the dark (BH) of the arena over the course of the 30-minute trial. Stars (*) indicate significances (** *p*<0.01; *** *p*<0.001).

As shown in Figure 6, 2 months post-bilateral-injection of AAV2-*ChR2V* into adult TKO mice, cells across the entire inner retina were transduced to express ChR2V (green). In Figure 6B the red cells are stained for β-galactosidase, the reporter gene that replaces the melanopsin gene in TKO mice. Interestingly, by double-labelling in this fashion we very rarely encountered β-galactosidase positive cells that had been transduced to express the microbial opsin (<5 cells per retina).

As shown in Figure 6C, following ChR2V transduction, TKO mice (denoted AAV2-*ChR2V* TKO) now show an aversion to light similar to that of WT mice (WTs spend 67±4% (mean±SEM) and AAV2-*ChR2V* TKOs spend 68±10% (mean±SEM) in the dark back half when the front half is illuminated). The AAV2-*ChR2V* TKO mice also exhibit a positive correlation in their behaviour over the duration of the trial (Figure 6D), spending most time in the dark BH at the end of the trial (74%). Two-way RM ANOVA comparing the untreated to AAV2-*ChR2V* treated TKOs reveals a significant effect of treatment (*p*<0.001), and a significant effect of time (*p*<0.05). Bonferroni post tests show that at all time points during the trial, AAV2-*ChR2V* TKOs are significantly more averse to light than the untreated animals in the light (Figure 6D). Importantly for addressing the role of pupillary constriction in BLA, the transduced mice exhibited this strong aversion to light in the absence of a detectable PLR (Figure S4).

## Discussion

The photopigment melanopsin has an established role in non-image forming behavioural responses to light such as circadian photoentrainment, negative masking and the induction of sleep [15], [28], [31], [32], [33]. It has also been shown to be sufficient for the acquisition of a Pavlovian association between light and impending negative reinforcement [34]. Here, using naïve adult mice, we confirm a new and important role for melanopsin in the attribution of emotional salience to light. Quite unexpectedly, our investigations also reveal a capacity for light perception/BLA in mice lacking three components deemed necessary for photoreception.

### Melanopsin mediates a slow behavioural aversion to light

As reported for normally sighted animals in previous studies, we found a clear aversion to light by WT mice within the first 5 minutes of our test. However, in MO mice, where melanopsin alone drives this response there is a slower, more gradual BLA over time. The majority of light:dark testing paradigms used to date employ short trials (5–10 min duration) and we suggest that this may be one factor in the failure to report light aversion in previous studies using retinal degnerate rodents [19], [20]. Our results are however consistent with those from a 22 h experiment suggesting a role for melanopsin in the preference displayed by *rd/*rod-ablated mice for a darkened nesting compartment [21]. Additionally, our data from MO mice shows that melanopsin-driven BLA has a strong positive correlation over time, potentiating light aversion over the course of our trial.

As with many other non-image forming responses to light, our data from MKO mice shows that melanopsin is not required for BLA to occur. However, these animals lack a positive correlation over time, a result implicating melanopsin in the potentiation of outer retinal signalling as this behaviour progresses. We also found that older MO mice lose the positive correlation in BLA across time due to an enhanced light aversion in the first five minutes, an intriguing finding that suggests increased potency of melanopsin signalling with advancing age in retinal degeneration.

Although we have not examined BLA in neonatal animals here, the age (8–14 days old) at which rats were used in the initial studies by Crozier and Pincus [1] strongly implicates the involvement of the melanopsin system. This is because outer retinal function does not contribute to retinal activation prior to postnatal day 10 in mice [59] and between postnatal days 12–14 in rats [60].

An important component of our behavioural paradigm is the direct comparison between behaviour in light versus complete darkness. This method takes advantage of a phenomenon whereby, in the absence of light, animals will choose to spend the majority of a 30 minute period in the front half of the arena. The incorporation of this behaviour into our data analysis may help to explain why light aversion has not been reported previously over short durations in retinally degenerate rodents.

The light:dark choice test is regarded as an unconditioned conflict paradigm, where the innate tendency for light avoidance conflicts with the propensity of mice to explore/escape novel places into which they are forced [6], [7]. To the best of our knowledge the robust behaviour in darkness we report has not previously been described. Although not easy to explain, we suggest this response may relate to an anxiety state which occurs in mice forced into a novel environment [61].

When mice are given the choice to freely explore something new they display a behaviour known as “neophobia” which involves initial retreat from and then progressive exploration of the novelty. If mice are presented simultaneously with a familiar and novel compartment to freely explore, they will spend approximately 75% of their time in the novel compartment. However, when they are forced into this novel compartment the animals display heightened anxiety levels, as measured by elevated corticosterone [61]. Thus, in the context of the present experiment, under complete darkness, a state of forced novelty exists. We suggest that in the absence of the aversive stimulus of light, mice may simply be returning to the point of entry which they associate with escape to the home cage.

In adult nocturnal rodents, BLA may contribute to diurnal behaviour by moving animals away from sunlight, towards darkened nesting areas where they would sleep. In this respect, the melanopsin-dependent temporal potentiation of BLA we identify here may be of particular importance in increasing motivation to leave open field environments. Given the role for melanopsin in modulating sleep [31], [32], [33] it will be important to examine the interaction between BLA and this other melanopsin-modulated behaviour in future experiments.

Although not available in the present study, it would be interesting to examine the performance of melanopsin aDTA mice [12], [32] in our paradigm, these mice will help to determine the extent to which the mRGC pathway is required for BLA. However, like the MKO mice reported here, mice with a targeted destruction of mRGCs retain a behavioural aversion to light [18], indicating that mRGCs are probably not an absolute requirement for BLA.

### Atropine application reveals a new element to light perception

Atropine pre-treatment led to a significant elevation of BLA in WT and MO mice, with both genotypes now responding to a similar level. The effect was particularly strong on the melanopsin component of BLA, strengthening the potentiation of this behaviour over time. In contrast, atropine failed to enhance the overall aversive response of MKO animals to light but did induce a positive correlation over time. We concluded from these experiments that BLA may normally be constrained by the PLR and that with fully dilated pupils, enhanced light stimulation of the retina increases the activity of outer and to a greater extent, inner retinal photoreceptors.

In order to control for the possibility that atropine may act independently of pupil dilation, we also added this drug to the eyes of TKO mice, which lack a PLR. To our complete surprise, this manipulation revealed an ability of TKOs to perceive light and display BLA. Previous work with these animals has shown that despite having an intact retina, they lack significant visual responsiveness [49]. Recent preliminary data indicates that TKO mice retain a small ERG with a spectral sensitivity matching rod opsin [54]. We report here that atropine application significantly enhances the b-wave component of this response and that bilateral axotomy abolishes the atropine-induced behavioural aversion to light exhibited by TKO mice. Thus, we suggest that atropine augments a residual retinal response in these animals, which is sufficient to drive BLA. In a complementary fashion, we confirm that in the absence of atropine, enhancing retinal light-responsiveness with ChR2 can also drive BLA in TKO mice in the absence of a PLR. The magnitude and temporal kinetics of this ChR2-mediated response are strikingly similar to BLA seen in WT mice, indicating that during development, the proximal neural circuitry for BLA can develop independently of normal rod, cone and melanopsin signalling. Interestingly, the behavioural effect in this experiment was achieved without significant transfection of mRGCs identified using β-galactosidase staining. Although this observation suggests that mRGCs may not be the only conduits mediating BLA, new evidence revealing an extended diversity of melanopsin expressing ganglion cells [36], [62] raises the possibility that ChR2V may have been expressed in mRGCs that we could not detect.

During the preparation of this manuscript we became aware of another study examining the ability of phototransduction deficient MO mice (Gnat1^−/−^, Cnga3^−/−^) to perform pattern discrimination [36]. While these mice fail to respond to visual gratings in an optokinetic tracking test, in a forced-swim test, they can learn to use gratings of low spatial frequency to predict positive reinforcement (presence of an escape platform), while control TKOs (*Opn4^−/−^*, *Gnat1^−/−^*, *Cnga3^−/−^*) cannot. This is accompanied by grating-induced c-Fos activation in primary visual cortex. The authors attribute these results solely to inner retinal melanopsin cells. Although no experimental data is presented, they also make the comment that control TKO mice retain an ability to discriminate between two screens in the visual learning task that vary significantly in luminance. This observation agrees with our findings of atropine-enhanced BLA and provides an independent account of light perception in TKO mice.

Interestingly, in another mouse model combining genetic ablation of rods with the disabling of cone phototransduction (*Rho^−/−^*, *Cnga3^−/−^*) there is no detectable ERG response [17]. This strongly implicates residual rod function in the retained visual capabilities of TKO mice, which have the same cone mutation (*Cnga3^−/−^*) but possess structurally intact rods (*Gnat1^−/−^*). This is in line with a previous report of atypical rod function under cone-isolating conditions [63] and cautions the use of genetic deactivation without cell death in order to isolate melanopsin function [32]. Indeed, it could be that because rods play an important role in rodent visual acuity within the photopic range [11], the melanopsin system may in fact be potentiating a rod-driven signal to allow pattern discrimination by the visual system of mice lacking both *Gnat1^−/−^* and *Cnga3^−/−^* [36].

In terms of a mechanism of action for atropine in enhancing light-mediated BLA, it seems likely that there is a combination of pupillary dilation to spatially increase retinal luminance and a direct action on retinal signal processing. Interestingly, this information may be processed in the brain independently of established nociceptive pathways, as atropine, applied topically to the eyes fails to alter light-evoked responses in the spinal trigeminal nucleus [43]. In line with our findings, another recent study also reports that atropine (in combination with phenylepherine) can enhance ERG b-wave amplitudes in C57 wildtype and retinal degenerate mice [64]. The ability of atropine to enhance retinal function has implications for the interpretation of data arising from a range of visual neuroscience studies that employ this drug prior to measuring visual function [12], [63], [65].

### Relevance of BLA in mice to human photophobia

Recently, the melanopsin system has been implicated in the circuitry by which light exacerbates the symptoms of migraine headache [42]. This study showed that patients with outer retinal degenerations consistently report migraine-associated photophobia. Using indirect evidence the authors propose a neural circuitry that involves information from mRGCs converging with trigemino-vascular signals in the lateral posterior thalamic nuclei before the integrated information is relayed up to cortical regions involved in pain processing.

Our results confirm that in retinal degenerate mice, melanopsin alone can drive a progressive behavioural aversion to light which is associated with activation of the visual and retrosplenial cortex. Both these structures are innervated to some extent by dura/light sensitive thalamic neurons [42]. In the context of behavioural aversion to light, our identification of melanopsin-driven c-Fos induction (Figure S1) in the retrosplenial cortex (RSC) is of particular interest because stimulation of this region in humans can cause autonomic responses linked to emotional processing [66]. The RSC is a posterior division of the cingulate cortex [67], a limbic structure which is active during the perception of photophobia in humans [44]. The established role of RSC in functions such as memory and navigation [68], together with its anatomical connectivity to structures such as the hippocampus and superior colliculus [69], [70] make this an important structure to examine in future studies exploring melanopsin's role in emotional and cognitive processing, which are widely regarded to be inter-linked [71].

### Conclusions

Melanopsin in isolation is capable of attributing emotional salience to light sufficient to produce an aversive behavioural response that potentiates over time. This finding has relevance to the understanding of how spatial movements may be integrated with diurnal sleeping patterns to control circadian behaviour. Given the potential role for melanopsin in human photophobia, the study of brain regions involved in assigning affective valence to luminance represents an interesting avenue for future research. Surprisingly, the use of atropine to examine the role of the PLR in BLA also revealed that light perception, sufficient to generate an aversive behavioural response can occur in TKO mice, lacking melanopsin, a PLR and proper rod/cone function. The re-instatement of BLA in ChR2-transfected TKO mice confirms that pupillary constriction is not a requirement for light aversion in rodents.

## Materials and Methods

### Animals

All procedures were conducted according to the Home Office (UK) regulations, under the Animals (Scientific Procedures) Act of 1986, and with local (UCL-Institute of Ophthalmology, London, UK) ethics committee approval.

Four types of mice were used, wildtype (WT), *rd/rd cl* (melanopsin only, MO) [14], [31], melanopsin knockout (MKO) *Opn4^−/−^* [30] and triple knockout (TKO) *Opn4^−/−^;Gnat1^−/−^;Cnga3^−/−^* [49]. The WT and MO are congenic on the C3H/He strain, whilst the MKO and TKO are a C57 BL6/129 mixed strain background. All animals were housed under a 12:12 light dark cycle, with food and water available *ad libitum*.

### Pupillometry

The PLR was measured in un-anaesthetised mice dark adapted for at least 1 hour. An infra-red light source was used to illuminate the left eye and frames were taken using a 12 bit SMD (1M60) digital camera mounted on top of a Leica MZ75 microscope using a magnification of 1. A long pass filter was interposed between the microscope lens and the mouse eye to block any light of less than 665 nm wavelength. The left eye was stimulated with broad-spectrum light originating from a xenon-arc lamp (Lambda DG-4, Linton Instrumentation) synchronized with the image capture using an electronic shutter (Melles-Griot). Short-pass and neutral density filters (Edmund Optics Ltd., York, UK) were combined to abolish stimulus light wavelengths above 600 nm. The light was then guided with a fibre optic through a light diffuser placed 2 cm away from the left eye stimulating with white light of 600 µW/cm^2^ irradiance. The eye was stimulated for 24 seconds while collecting spatially binned (2×2) frames of the eye at 4 Hz. The pupil area was estimated off-line at each frame by an observer using customized MATLAB software and the results were downsampled to 1 Hz. Pupillometry was carried out on n = 5 TKO, n = 3 MO, and n = 4 TKO mice treated with AAV-*ChR2V*. One day after PLR assessment of the TKO and MO the same mice had bilateral application of atropine sulphate, 1%, (Minims, preservative free) under dim red light. After 1 hour of dark adaptation recording of their PLR was made as described above.

### Testing of open field light aversion behaviour

Adult mice (∼100–250 days of age) of mixed sexes were used. We chose to use mice naïve to the experimental arena because although habituation is used in some light/dark choice protocols [72] this can reduce the amount of time spent in the dark [73] which may mask subtle light responses. The open field arena is shown in Figure 1A. The arena is square (26×26 cm) and is divided in half into an open front-half (FH) and an enclosed back-half (BH), with a small door through which the mouse can enter the enclosed area. The FH of the arena was either illuminated (light FH) or remained in darkness (dark FH) with a light-impervious cloth used to baffle the arena from stray sources of light. White light illumination was provided by a Philips Energy Light (Philips, Guildford UK) suspended 0.75 m above the whole arena (irradiance at floor level 600 µW/cm^2^ or ∼1300 Lux). The illumination did not cause a measurable change in temperature in the FH of the arena compared to the BH, as measured using heat probes. Air conditioning in the room served to regulate the temperature and also produced background white noise.

Only naïve animals were tested, that did not have previous exposure to the arena. All tests were carried out during the light phase (ZT1-11) of their light:dark cycle, and all animals were light adapted. At the start of each trial, each mouse was placed in the FH of the arena under dim red illumination (after which this red light was turned off) and left for 30 minutes with either illumination or darkness in the FH. The time spent in each compartment was monitored using TRUSCAN (Coulburn Instruments, Inc Allentown, PA). After each trial the arena was thoroughly washed and then wiped with 70% ethanol and dried.

Some animals were treated with atropine prior to being tested in the arena. Here, animals were taken from their holding room, to a separate procedure room where 1 drop of atropine sulphate, 1%, (Minims, preservative free) was applied bilaterally, and the animals were then left in their home cage for at least 0.5–2 hours prior to being tested in a separate procedure room containing the open field arena.

Animals that did not enter the BH of the arena within the first 5 minutes of a trial were discounted from the analysis. Total numbers tested and those discounted are shown in table S1. In general, most animals entered the BH within the first couple of minutes and, in terms of latency to enter the BH, no significant differences between the genotypes or between different illuminations (light vs. darkness) were found (data not shown).

### Bilateral intraocular axotomy procedure

For axotomy surgery, triple knockout mice were deeply anaesthetized with a mixture of medetomidine hydrochloride (1 mg/kg) (Domitor, Pfizer, Kent, UK) and ketamine (75 mg/kg) and placed securely in a nose bar with eyes covered in ViscoTears (Novartis Pharmaceuticals UK Ltd). An ophthalmic operating microscope (Olympus) was used to visualize the optic nerve head directly through a glass coverslip before gripping the extra-ocular muscles with a pair of fine-toothed microsurgical tweezers (FST) and inserting a 30-gauge needle (attached to a 2.5 µl Hamilton syringe) through the sclera, directly into the sub-retinal space. This technique uses the same needle and sub-retinal approach that is routinely employed in cell transplantation studies [74]. Once the needle was sub-retinal and adjacent to the optic nerve head, the optic nerve (together with the central retinal artery) was easily severed using a swift back and forth movement. This surgical procedure is summarized in Figure 5A (in a schematic adapted from May and Lutjen-Drecoll, 2002 [75]). At the time of surgery, axotomy was confirmed by injecting 2 µl of saline to produce a retinal detachment beneath the successfully severed optic nerve head.

Following bilateral axotomy surgery, all animals were given an intra-peritoneal injection of the analgesic carprofen 5 mg/kg (Rimadyl, Pfizer, Kent, UK) and recovered with the anaesthetic antidote atipamezole 0.5 mg/kg (Anti-sedan Pfizer, Kent, UK). Animals had recovered well by the following morning and were run in the light aversion assay 8 days post-surgery. One day following the completion of behavioural testing, all mice were perfused and their brains processed for calretinin immunohistochemistry. In addition to the axotomised mice, for the purposes of comparison, several age-matched untreated TKOs (n = 3) were also perfused and their brains processed for calretinin immunohistochemistry. The anatomical positioning of brain sections was determined using the mouse brain atlas [76]. The calcium binding protein calretinin is expressed ubiquitously in retinal ganglion cell axons and is a well-characterised marker for assessing deafferentation of subcortical retino-recipient structures in the rodent [77]. Previous work in the rat [78] and mouse [79] has demonstrated that calretinin-positive axonal fibres in the superficial layers of superior colliculus originate exclusively from retinal ganglion cells and that these fibres are lost 7 days following successful optic nerve section.

### AAV vector injection

The preparation of the adeno-associated viral (AAV) vector used here has been described in detail previously [57]. In brief, it contains a *Channelrhodopsin-2/Venus* (*ChR2V*) fusion gene under the control of a hybrid cytomegalovirus/chicken β actin promoter. Four TKO adult mice (162 day-old) underwent bilateral intra-vitreal injections of the AAV2-*ChR2V* viral vector suspension (1×10^12^ particles/ml, measured by an ELISA assay as described previously [80]).

Mice were anaesthetised as above and the head stabilised in a nose-bar before inserting a 30-gauge needle (attached to a 2.5 µl Hamilton syringe) into the vitreous cavity. A total volume of 2 µl of the viral vector suspension was injected into each eye, followed by a parasentesis counter-injection made below the *ora serrata* (to relieve intraocular pressure). The mice were recovered as described above and tested two-months post-surgery for photophobic behaviour and pupillometry. Finally, they were perfused and the eyes processed for immunohistochemistry, with one retina from each animal removed and processed as a flat mount whilst the other was cryprotected, frozen and sectioned.

### Electroretinogram recordings

Experimentation was performed under dim red light (<0.25 µW/cm^2^, >650 nm), and mice were long-term dark adapted (>12 hr) prior to recording. To compare the effects of atropine on the TKO ERG, mice were divided into two groups: 5 mice received atropine sulphate eye drops (1%; minims, preservative free) in each eye 30 minutes prior to recording, and 5 mice received no drops. Mice were initially anaesthetised with intra-peritoneal ketamine (70 mg/kg) and xylazine (7 mg/kg), which was maintained with an injection of subcutaneous ketamine (72 mg/ml) and xylazine (5 mg/ml).

Hypromellose solution (0.5%; Alcon Laboratories, Ltd., UK) was applied to each eye to retain corneal moisture and to provide sufficient adherence of a contact lens electrode to the corneal surface. A silver wire bite bar provided head support and acted as a ground, and a needle reference electrode (Ambu® Neuroline) was inserted approximately 5 mm from the base of contralateral eye, sufficiently distal to exclude signal interference. Electrodes were connected to a Windows PC via a signal conditioner (Model 1902 Mark III, CED, UK), which differentially amplified (x3000) and filtered (band-pass filter cut-off 0.5 to 200 Hz) the signal, and a digitizer (Model 1401, CED). Throughout experimentation, core body temperature was maintained at ∼37°C via a homeothermic heat mat (Harvard Apparatus, Kent, UK). For ten minutes prior to first recordings, electrode stability was monitored; electrodes displaying any baseline instability were rejected.

A xenon arc source (Cairn Research Ltd., Kent, UK) connected to a ganzfeld sphere provided white light flashes with a peak corneal irradiance of 1.58 mW/cm^2^ (2370 Lux). A series of 15 ms flashes were applied using an electrically controlled mechanical shutter (Cairn Research Ltd.) with a 40 s interstimulus interval. An average ERG response was generated from 25 flashes, and the b-wave amplitude measured (from a-wave peak to b-wave peak) and compared statistically.

### Immunohistochemistry

Animals were deeply anaesthetised with sodium pentobarbital (60 mg/kg) and then perfused with 0.1 M PBS followed by 4% paraformaldehyde (in 0.1 M phosphate buffer), with overnight post-fixation at 4°C. Tissues to be cryostat sectioned were cryoprotected overnight at 4°C in 30% sucrose solution (in 0.1 M PBS), and then frozen with a dry ice/acetone slurry. Coronal brain sections (30 µm thick) were cut on the cryostat and processed free-floating, while retinal sections were cryosectioned (14 µm thick) and mounted onto Superfrost Plus slides (BDH, Poole, UK).

Tissues were blocked for 2 h with 5% normal donkey serum (NDS) in PBS containing 0.3% (retinal/brain sections) or 3% (flat mounts) Triton X-100 (PBS-TX). The tissue was subsequently incubated overnight in PBS-TX containing 1% NDS and either a goat primary antibody raised against calretinin (1∶1000, Swant, Bellinzona, Switzerland) or a rabbit anti-β galactosidase antibody (1∶5,000, Abcam, Cambridge, UK). Following washes in PBS, tissue was incubated for 2 h in PBS-TX containing 2% NDS and an appropriate TRITC-labelled secondary antibody (1∶200, Jackson ImmunoReseach, West Grove, PA). Tissue was washed extensively in PBS and TRIS buffer. Cell nuclei were counterstained with DAPI (1∶5,000 Sigma) before cover-slipping with Vectashield (Vector Laboratories, Burlingame, CA). Fluorescence labelling was examined using a Zeiss confocal microscope (with LSM Image Browser software, Welwyn Garden City, UK).

### Statistical Analysis

All data was analysed using GraphPad Prism software (GraphPad Software, San Diego, CA). Prior to analysis by Student's *t*-tests or ANOVA the proportional light aversion data were transformed Y = Arcsine(Y). One-tailed Student's *t*-tests were used to analyse both the effect of light on the total amount of time spent in the dark BH (light FH versus dark FH) over the whole 30-minute trial, and also the electroretinogram b-wave data. To analyse the effect of rods and cones, light aversion behaviour was compared between the congenic WT and MO by two-way ANOVA (factors: genotype and light) followed by Bonferroni's multiple comparison tests. To analyse the effect of atropine a two-way ANOVA (factors: light and atropine) was performed followed by Bonferroni's multiple comparison tests. The effect of light over the duration of the trial was investigated by using both regression analysis and two-way repeated measures ANOVA (RM ANOVA), factors: light and duration of the trial, (subjects were significantly matched in all cases *p*<0.0001) this was followed by Bonferroni post-tests with light FH versus dark FH at each time point in the trial.

## Supporting Information

Figure S1 **Light induced c-fos in the visual and retrosplenial cortex (RSC) of MO (*rd/rd cl*) mice.** Images on the left are from an animal that remained in the dark whilst those on the right from an animal that was exposed to light. Nuclei positive for the immediate early gene c‐Fos are green, whilst neurofilament‐H (NF‐H) is in red. (**A**) Montage of the cortex, −3.52 mm from the Bregma, Scale bar 400 µm. (**B**) Higher magnification of the medial visual cortex (V1/2) clearly showing light induced neural activity in layers II–VI. (**C**) Higher magnification of the retroplenial cortex showing light induced c‐Fos, (**B**–**C**) Scale bars 200 µm. Methods: Mice (n=3 per condition) were dark‐adapted overnight and at 07:00, still in their home cages, either exposed to 1.5 hours of ∼1300 lux white light or maintained in darkness. They were then perfused and brain sections processed for immunohistochemistry as described in the main text using rabbit anti‐c‐fos (PC38, Calbiochem, 1∶5,000) and mouse anti‐neurofilament heavy chain (SMI‐32, Covance, 1∶5,000) followed by secondaries antibodies (FITC anti‐rabbit IgG and TRITC anti‐mouse IgG, both from Jackson ImmunoReseach, West Grove, PA). The neurofilament‐H antibody was used to match up sections using cytoarchitectural boundaries in the cortex, as described previously by Van der Gucht *et al*., 2007
(TIF)

Figure S2 **Behavioural light aversion in old versus young MO (*rd/rd cl*) mice.** (**A**) The amount of time old animals (394±46 day-old; mean±SD) spend in the dark back-half (BH) during the 30-min trial is not significantly different to the amount of time spent there by younger animals (166±6 day-old), although the average time that the old animals spend in the dark is slightly higher (, 58% versus, 46%). (**B**) Over the course of the trial it is revealed that the old animals spend significantly more time (, ∼70%) in the dark than the younger animals during the first 5 minutes of the trial (Two-way repeated measures ANOVA demonstrates: (1) a significant interaction (p<∼0.05) aging X duration of the trial and (2) a significant effect of duration (p<0.05), Bonferonni post-tests show that in the first 5 minutes the old animals spend significantly more time in the dark (p<0.01) than younger animals). It seems unlikely that this is due to poorer mobility in the old animals as they continued to move around the arena sampling both light and dark regions for the rest of the trial. Due to this behaviour during the first 5-minutes there is no longer a significant positive correlation of photophobic behaviour in the old animals, they continue spending a similar proportion of their time in the dark BH throughout the 30 minutes. Abbreviations: BH, back-half; MO, melanopsin only.
(TIF)

Figure S3 **Calretinin positive retinal-afferents (red) are lost 9 days post axotomy in the olivary pretectal nucleus (OPT) and the optic chiasm (och) of TKO mice.** Compare **A** with **B** for the OPT and **C** with **D** for the och. Brain sections from equivalent Bregma positions were imaged in control and axotomised brains as indicated in **A** for the OPT and in **C** for the och. Scale bar in D for all plates is 100 µm. Abbreviations: oc, optic chiasm; OPT, olivary pretectal nucleus; TKO, triple knockout.
(TIF)

Figure S4 **Pupillometry in triple knockout (TKO) mice following transduction of the inner retina with Channelrhodopsin 2/Venus fusion protein.** At two-months post-introduction of the AAV2-*ChR2V* the pupillary light reflex has not been re-instated in these animals.
(TIF)

Table S1 (DOC)
