# Supplementary figures and images for: Dissecting a Role for Melanopsin in Behavioural Light Aversion Reveals a Response Independent of Conventional Photoreception
Source: PLoS One. 2010 Nov 29;5(11):e15009. doi: 10.1371/journal.pone.0015009 (PMC2993953; doi:10.1371/journal.pone.0015009)

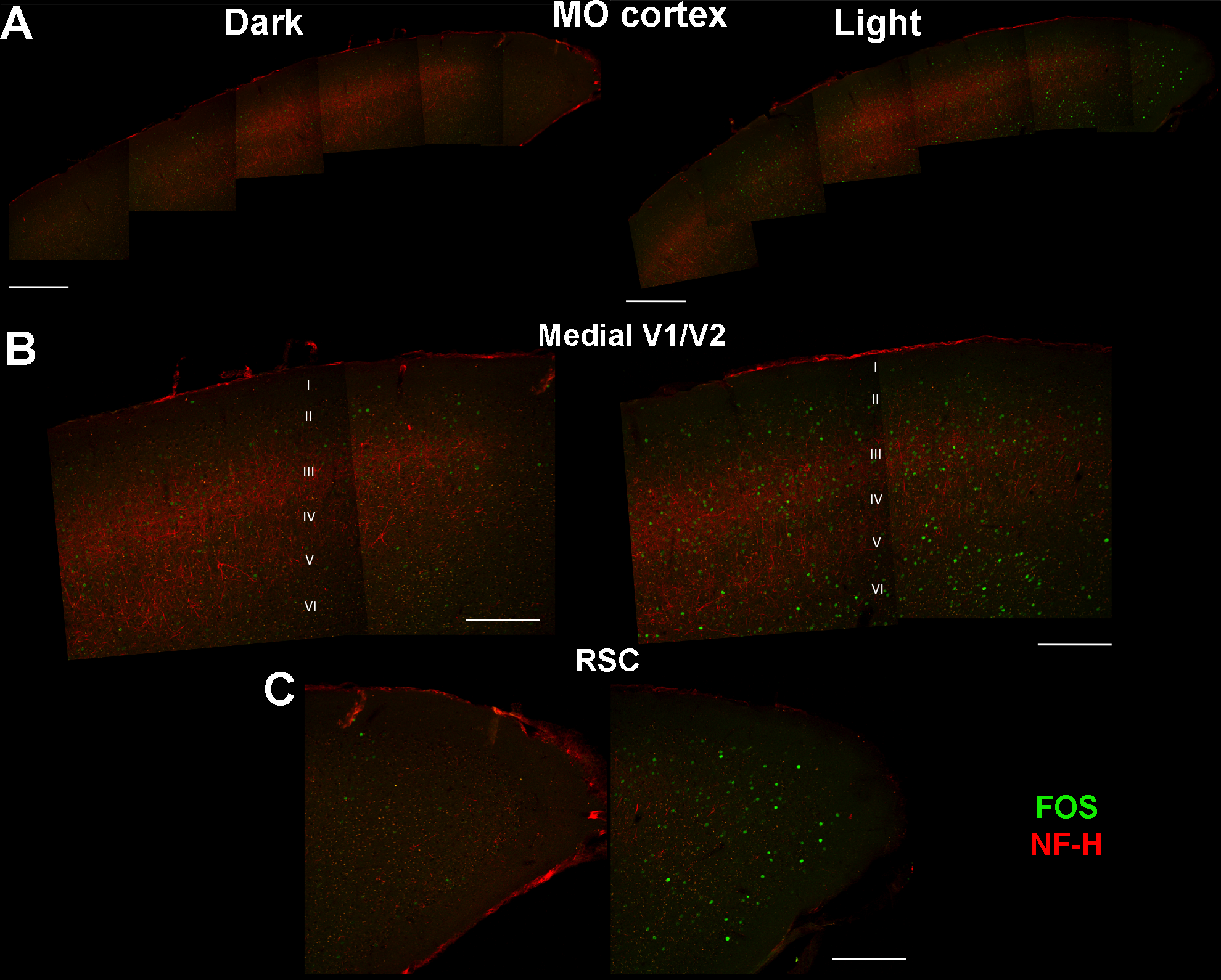

Supplement: Figure S1 — Light induced c-fos in the visual and retrosplenial cortex (RSC) of MO (rd/rd cl) mice. Images on the left are from an animal that remained in the dark whilst those on the right from an animal that was exposed to light. Nuclei positive for the immediate early gene c‐Fos are green, whilst neurofilament‐H (NF‐H) is in red. (A) Montage of the cortex, −3.52 mm from the Bregma, Scale bar 400 µm. (B) Higher magnification of the medial visual cortex (V1/2) clearly showing light induced neural activity in layers II–VI. (C) Higher magnification of the retroplenial cortex showing light induced c‐Fos, (B–C) Scale bars 200 µm. Methods: Mice (n=3 per condition) were dark‐adapted overnight and at 07:00, still in their home cages, either exposed to 1.5 hours of ∼1300 lux white light or maintained in darkness. They were then perfused and brain sections processed for immunohistochemistry as described in the main text using rabbit anti‐c‐fos (PC38, Calbiochem, 1∶5,000) and mouse anti‐neurofilament heavy chain (SMI‐32, Covance, 1∶5,000) followed by secondaries antibodies (FITC anti‐rabbit IgG and TRITC anti‐mouse IgG, both from Jackson ImmunoReseach, West Grove, PA). The neurofilament‐H antibody was used to match up sections using cytoarchitectural boundaries in the cortex, as described previously by Van der Gucht et al., 2007 (TIF) [file pone.0015009.s001.tif]

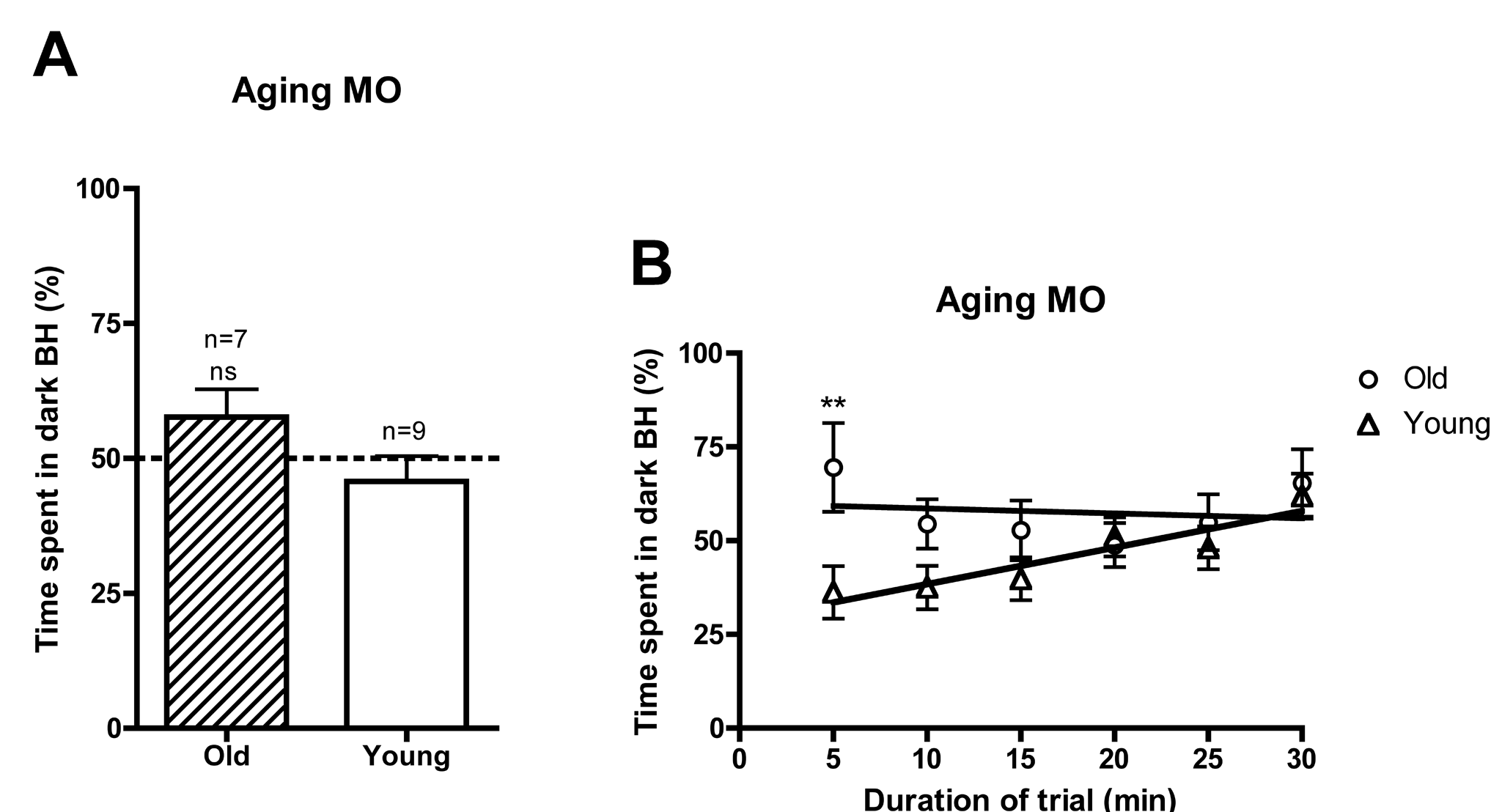

Supplement: Figure S2 — Behavioural light aversion in old versus young MO (rd/rd cl) mice. (A) The amount of time old animals (394±46 day-old; mean±SD) spend in the dark back-half (BH) during the 30-min trial is not significantly different to the amount of time spent there by younger animals (166±6 day-old), although the average time that the old animals spend in the dark is slightly higher (, 58% versus, 46%). (B) Over the course of the trial it is revealed that the old animals spend significantly more time (, ∼70%) in the dark than the younger animals during the first 5 minutes of the trial (Two-way repeated measures ANOVA demonstrates: (1) a significant interaction (p<∼0.05) aging X duration of the trial and (2) a significant effect of duration (p<0.05), Bonferonni post-tests show that in the first 5 minutes the old animals spend significantly more time in the dark (p<0.01) than younger animals). It seems unlikely that this is due to poorer mobility in the old animals as they continued to move around the arena sampling both light and dark regions for the rest of the trial. Due to this behaviour during the first 5-minutes there is no longer a significant positive correlation of photophobic behaviour in the old animals, they continue spending a similar proportion of their time in the dark BH throughout the 30 minutes. Abbreviations: BH, back-half; MO, melanopsin only. (TIF) [file pone.0015009.s002.tif]

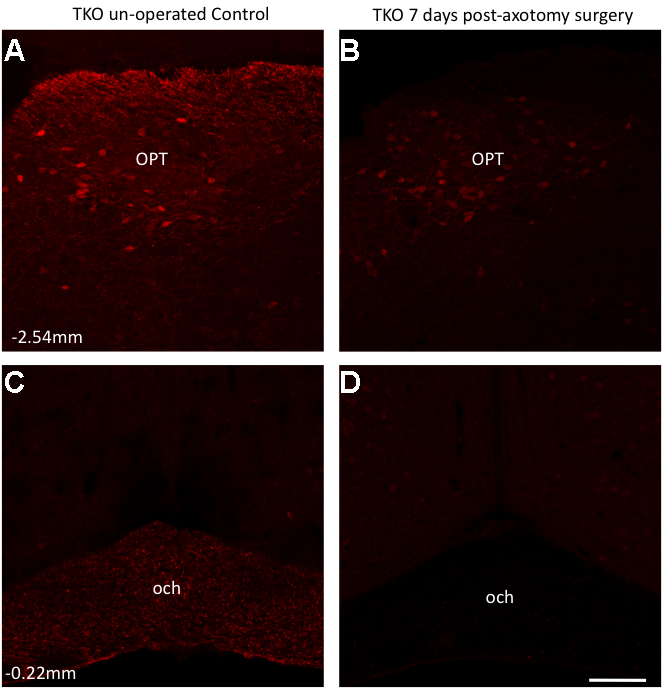

Supplement: Figure S3 — Calretinin positive retinal-afferents (red) are lost 9 days post axotomy in the olivary pretectal nucleus (OPT) and the optic chiasm (och) of TKO mice. Compare A with B for the OPT and C with D for the och. Brain sections from equivalent Bregma positions were imaged in control and axotomised brains as indicated in A for the OPT and in C for the och. Scale bar in D for all plates is 100 µm. Abbreviations: oc, optic chiasm; OPT, olivary pretectal nucleus; TKO, triple knockout. (TIF) [file pone.0015009.s003.tif]

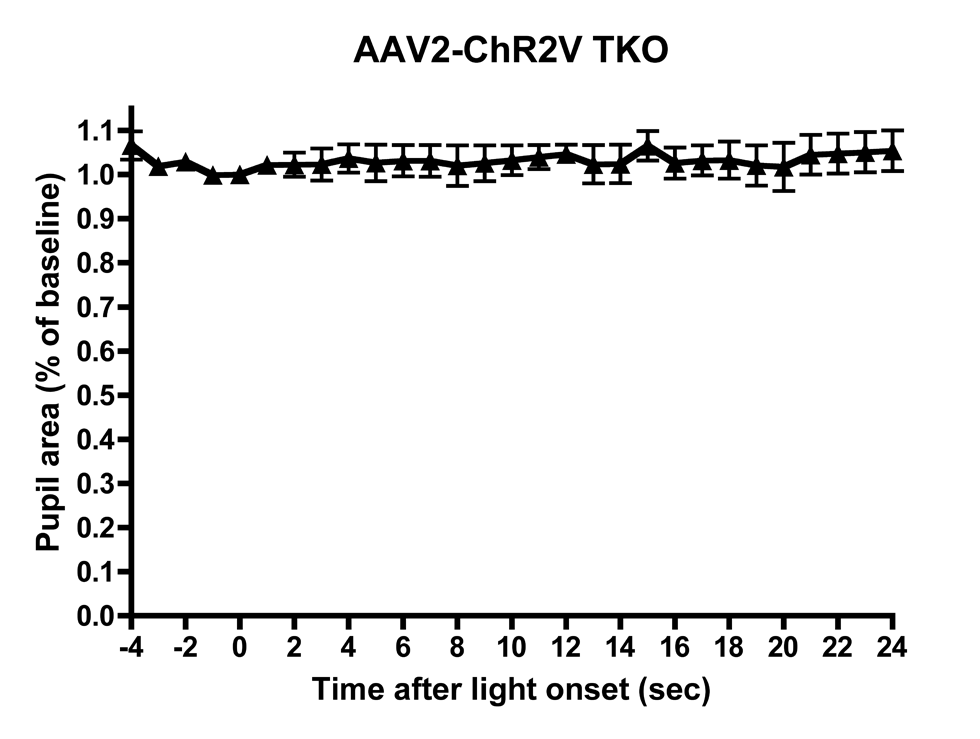

Supplement: Figure S4 — Pupillometry in triple knockout (TKO) mice following transduction of the inner retina with Channelrhodopsin 2/Venus fusion protein. At two-months post-introduction of the AAV2-ChR2V the pupillary light reflex has not been re-instated in these animals. (TIF) [file pone.0015009.s004.tif]
