# Supplementary material for: Dissecting a Role for Melanopsin in Behavioural Light Aversion Reveals a Response Independent of Conventional Photoreception
Source: PLoS One. 2010 Nov 29;5(11):e15009. doi: 10.1371/journal.pone.0015009 (PMC2993953; doi:10.1371/journal.pone.0015009)
Supplement: Table S1 — (DOC) [file pone.0015009.s005.doc]

**Table S1** Total numbers of animals tested for behavioural light aversion

| **Genotype** | **Treatment** | **Animals tested (N)** | **Discounted ^a^** |
| --- | --- | --- | --- |
| WT | Light Untreated | 11 | 2 |
| WT | Dark Untreated | 10 | 0 |
| MO | Light Untreated | 10 | 1 |
| MO | Dark Untreated | 10 | 0 |
| MKO | Light Untreated | 11 | 1 |
| MKO | Dark Untreated | 6 | 0 |
| TKO | Light Untreated | 11 | 1 |
| TKO | Dark Untreated | 5 | 0 |
| WT | Light Atropine | 9 | 3 |
| WT | Dark Atropine | 12 | 1 |
| MO | Light Atropine | 14 | 4 |
| MO | Dark Atropine | 11 | 4 |
| MKO | Light Atropine | 13 | 4 |
| MKO | Dark Atropine | 11 | 2 |
| TKO | Light Atropine | 11 | 0 |
| TKO | Dark Atropine | 10 | 0 |
| TKO | Light Axotomy/Atropine | 11 | 0 |
| TKO | Dark Axotomy/Atropine | 11 | 0 |
| TKO | Light AAV2-ChR2V | 4 | 0 |

^a^ Animals that did not enter the back-half of the arena within the first 5 minutes of the trial were discounted from the analysis.
